# Investigating Neuroprotective Effects of Berberine on Mitochondrial Dysfunction and Autophagy Impairment in Parkinson’s Disease

**DOI:** 10.3390/ijms26157342

**Published:** 2025-07-29

**Authors:** Hae-Rim Cha, Jin-Seok Kim, Jin-Hyeob Ryu, Hyun-Jeong Cho

**Affiliations:** 1 Department of Biomedical Laboratory Science, College of Medical Science, Konyang University, 158 Gwanjeodong-ro, Seo-gu, Daejeon 35365, Republic of Korea; 22804522@konyang.ac.kr (H.-R.C.); wlstjr1206@naver.com (J.-S.K.); 2 BIORCHESTRA Co., Ltd., 1 Gukjegwahak 2-ro, Yuseong-gu, Daejeon 34000, Republic of Korea; branden.ryu@biorchestra.com

**Keywords:** Parkinson’s disease, natural product, berberine, mitochondria dysfunction, autophagy impairment

## Abstract

Parkinson’s disease (PD) is a common neurodegenerative disorder with substantial global impact. Although current therapies can provide symptomatic relief, they are often associated with high costs and adverse effects. Natural compounds with a history of traditional medicinal use have emerged as promising alternatives. In this study, we investigated the therapeutic potential and underlying mechanisms of berberine in both cellular and animal models of PD. In vitro, SH-SY5Y cells exposed to 6-hydroxydopamine (6-OHDA) exhibited decreased viability and increased oxidative stress, both of which were significantly alleviated by berberine treatment based on cell viability assays and DCFH-DA staining. Western blot analysis revealed that berberine modulated the AMPK–PGC-1α–SIRT1 signaling pathway and restored the expression of autophagy-related proteins LC3B and P62, suggesting that berberine could improve mitochondrial function and autophagy balance. In vivo studies using a 6-OHDA-induced PD mouse model further confirmed these effects, showing that berberine could improve motor function and lead to molecular changes consistent with in vitro studies. Additionally, safety evaluations indicated no significant hepatotoxicity based on AST and ALT levels. Body weight also remained stable throughout treatment. Collectively, our findings suggest that berberine can not only alleviate PD-related symptoms but also target key pathological mechanisms, supporting its potential as a therapeutic candidate for PD and other neurodegenerative diseases.

## 1. Introduction

Parkinson’s disease (PD) is the second most common neurodegenerative disease. The prevalence of PD increases with age. The lifetime risk of PD is 2.0% for men and 1.3% for women [1]. Neuropathological changes associated with PD include α-synuclein-containing Lewy bodies and an irreversible loss of dopaminergic neurons in the substantia nigra pars compacta (SNpc) [2,3]. To establish models that mimic PD in vitro and in vivo, 6-hydroxydopamine (6-OHDA), a neurotoxic compound, is often used to induce neurotoxicity and oxidative stress [4,5]. Recently, oxidative stress has been regarded as an essential factor that can result in the dysfunction or death of neuronal cells [6,7] by causing mitochondrial dysfunction and autophagy impairment [8,9,10,11].

Mitochondria are dynamic organelles with numerous functions. In addition to their role in energy generation, they are closely involved in calcium homeostasis, stress response, and cell death pathways. Mitochondrial dysfunction is known to lead to cellular damage. It is associated with neurodegenerative diseases such as PD. Mitochondria can be targets of reactive oxygen species (ROS). Captivation evidence has suggested that mitochondrial dysregulation plays a crucial role in the pathogenesis of PD [12]. MPTP (N-methyl-4-phenyl-1,2,3,6-tetrahydro), its metabolite MPP+, rotenone, and 6-OHDA, a non-selective oxidant, are known to exert their toxicities by inhibiting the mitochondrial electron transport complex (ETC) [13,14,15]. The brain consumes about 20% of the oxygen supplied to the body [16]. When mitochondrial ETC I is damaged, ROS is excessively generated during the process of oxygen processing. An increased ROS level is a central mechanism for the onset of dopaminergic neuronal death [17]. Furthermore, accumulation of reactive cellular ROS associated with mitochondrial dysfunction can cause modifications of macromolecules such as proteins and nucleic acids. Protein oxidation can induce activity loss and/or protein unfolding along with a tendency to promote intracellular and extracellular protein oligomerization and aggregation [18].

Autophagy is one of the major catabolic quality control mechanisms. It is adapted for the degradation of soluble as well as large and insoluble cytosolic materials such as aggregated proteins and damaged organelles [19,20,21]. There are three types of autophagy, each with a unique mechanism for delivering substrates to lysosomes. These three types are micro-autophagy, chaperone-mediated autophagy (CMA), and macro-autophagy. Defects in macro-autophagy (often referred to as autophagy) are common features of human diseases, including neurodegenerative diseases [22]. In neurons, autophagy is essential for the degradation of neurotoxic factors such as alpha-synuclein, which is pathogenic in PD, and damaged organelles that are selected by ubiquitylation and recognized by the autophagic machinery [23]. If misfolded proteins such as a-synuclein accumulate due to autophagic–lysosomal dysfunction, defective mitochondria cannot be degraded. Instead, they will accumulate, generating ROS that can damage healthy mitochondria in the vicinity. Thus, mitochondrial health and autophagy balance are closely related to each other. When a disorder such as PD occurs, it can lead to a vicious cycle in which the consequences of the disease further contribute to its progression [24,25,26].

In eukaryotic cells, AMP-activated protein kinase (AMPK), an enzyme that plays an important role in maintaining cellular energy homeostasis, is a sensor that detects AMP/ATP ratio within cells. It is a Ser/Thr kinase that is activated when the AMP/ATP ratio changes [27]. AMPK promotes mitochondrial biogenesis through the growth and division of existing mitochondria. Specifically, it can be regulated by peroxisome proliferator-activated receptor γ coactivator 1α (PGC-1α) and Sirtuin 1 (SIRT 1) in downstream pathways. When AMPK is activated, it increases the expression of PGC-1a through direct phosphorylation [28,29], while SIRT mainly affects mitochondrial function through two existing pathways: a PGC-1α-dependent pathway and a PGC-1α-independent pathway [30]. In the PGC-1α-dependent pathway, SIRT1 plays a role in activating PGC-1α through deacetylation, which is important for energy metabolism [31,32,33]. SIRT 1 activates AMPK. Activated AMPK can upregulate the expression of SIRT 1. Therefore, cellular energy or metabolic stress can lead to mitochondrial fragmentation, which can result in cell death in severe cases. The AMPK-SIRT1-PGC-1α axis pathways can also influence autophagy, contributing to the maintenance of neuronal homeostasis [34].

Berberine is an isoquinoline alkaloid isolated from roots, barks, and rows of several types of medicinal plants such as *Hydrastis canadensis*, *Berberis aristata*, *Coptis chinensis*, *Coptis rhizome*, *Coptis japonica*, *Phellondendron amurense*, and *Phellondendron chinense schneid*. Numerous research studies have demonstrated different pharmacological and therapeutic effects of berberine, including anxiolytic, analgesic, anti-inflammatory, antipsychotic, antidepressant, and anti-amnesic properties [35,36]. One study has shown that the induction of HO-1 in SH-SY5Y cells can protect against 6-OHDA-induced human dopaminergic neuronal cell death [37]. In an animal experiment, oral administration of berberine inhibited cell death in the hippocampus and attenuated dopaminergic neurodegeneration, leading to improved memory and increased motor balance [38]. Berberine is also known as an activator of AMPK [39,40,41].

Although previous studies have demonstrated the neuroprotective properties of berberine and its role as an AMPK activator [39,40,41], its specific involvement in regulating mitochondrial function and autophagy through the AMPK–SIRT1–PGC-1α signaling axis in PD models remains poorly understood.

The present study aimed to investigate the ability of berberine to attenuate mitochondrial dysfunction and autophagy impairment through the AMPK-PGC-1α-SIRT1 axis via its antioxidant effects. Expression levels of each protein in 6-OHDA-induced SH-SY5Y cells and C57BL/6 mouse PD models were compared, and the efficacy of berberine was evaluated in behavioral experiments.

## 2. Results

### 2.1. Berberine Protects SH-SY5Y Cells Against 6-OHDA-Induced Neurotoxicity

**Figure 1:**
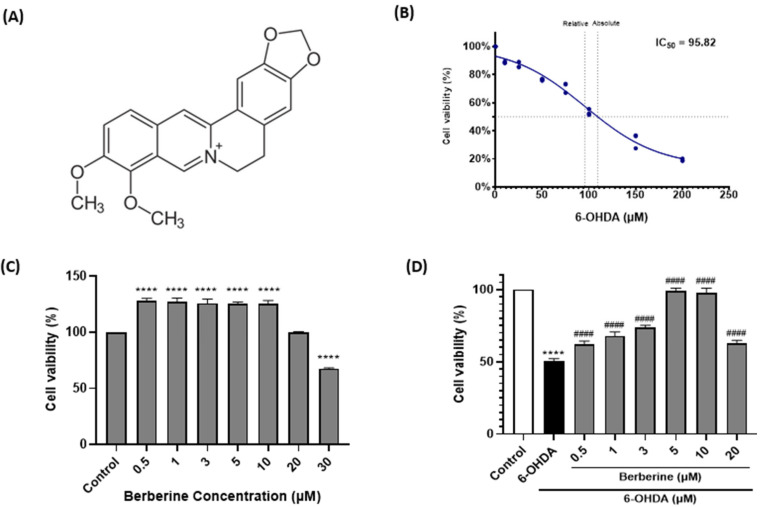
Berberine alleviates effect of 6-OHDA on cell viability in SH-SY5Y cells. (**A**) Chemical structure of berberine. (**B**) Cell viability after treatment with 6-OHDA at various concentrations (10, 25, 50, 75, 100, 150, 200 µM) for 6 h. (**C**) Cell viability after treatment with berberine at various concentrations (0.5, 1, 3, 5, 10, 20, 30 µM) for 24 h. (**D**) Cell viability after pretreatment with berberine at various concentrations (0.5, 1, 3, 5, 10, 20 μM) for 24 h followed by treatment with 6-OHDA (100 μM) for 6 h. Cell viability was measured using MTT method. Similar results were obtained from five independent experiments. Data are presented as mean ± SD (one-way ANOVA followed by Turkey’s post hoc test). ****, *p* < 0.001 compared to the control group; ^####^ *p* < 0.001 compared to the 6-OHDA-only treatment group.

6-OHDA, a neurotoxic compound, can lead to neurotoxicity, oxidative stress, and mitochondrial dysfunction. To explore the potential neuroprotective effect of berberine (Figure 1A), we used 6-OHDA to induce neurotoxicity in SH-SY5Y cells. The IC_50_ value of 6-OHDA in SH-SY5Y cells was determined by treating cells with various concentrations of 6-OHDA followed by cell viability assessment. The IC_50_ value represents the concentration of 6-OHDA at which 50% of the cell viability is inhibited. Based on our experimental results, the IC_50_ of 6-OHDA in SH-SY5Y cells was calculated to be 95.82 µM (Figure 1B). This indicates that at this concentration, 6-OHDA could inhibit 50% of the viability of SH-SY5Y cells. As shown in Figure 1C, treatment of SH-SY5Y cells with berberine at concentrations less than 30 µM for 24 h did not cause much toxicity. However, 30 µM of berberine decreased cell viability. Therefore, the appropriate concentration of berberine for treating cells was set at under 20 µM. When cells were treated with 6-OHDA at various concentrations, the IC_50_ value was found to be approximately 100 µM, which resulted in significant cell death. However, when cells were pre-treated with berberine for 24 h and subsequently exposed to 100 µM 6-OHDA for another 6 h, cell death caused by 6-OHDA was gradually alleviated (Figure 1D).

### 2.2. Berberine Decreases ROS Production in 6-OHDA-Treated SH-SY5Y Cells

**Figure 2:**
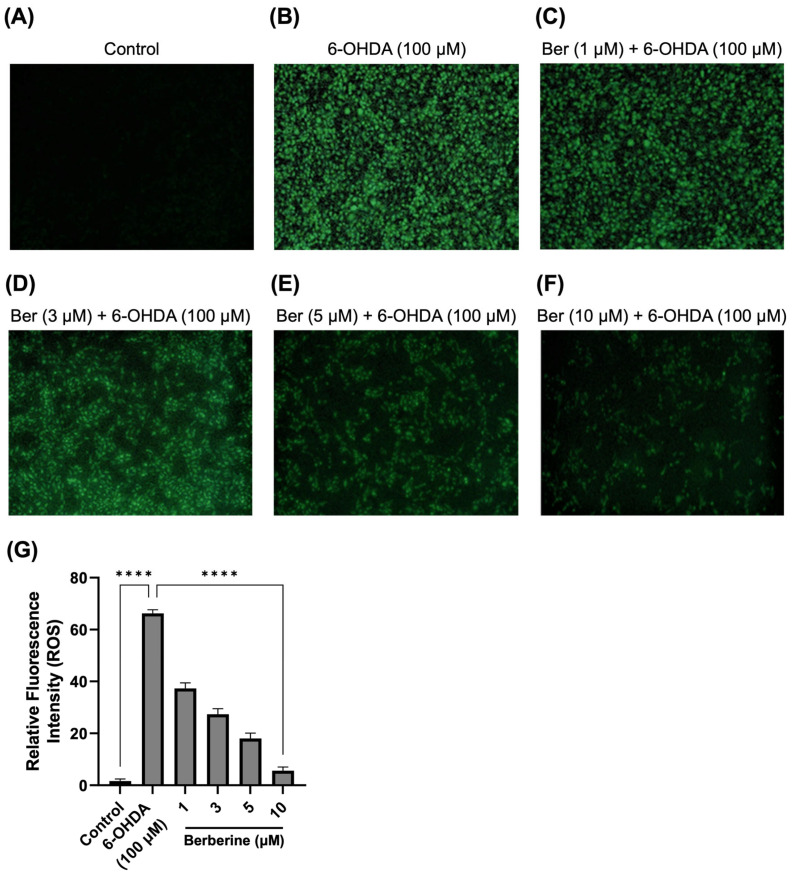
Effect of berberine on production of ROS. (**A**) Control group. (**B**) 6-OHDA-only group. (**C**) Berberine (1 µM) pretreatment + 6-OHDA group. (**D**) Berberine (3 µM) pretreatment + 6-OHDA group. (**E**) Berberine (5 µM) pretreatment + 6-OHDA group. (**F**) Berberine (10 µM) pretreatment + 6-OHDA group. (**G**) Statistical analysis of the relative fluorescence intensity of ROS. DCFH-DA staining results were obtained after pretreating cells with berberine at various concentrations (1, 3, 5, 10 μM) for 24 h, followed by treatment with 6-OHDA (100 μM) for 6 h. The intensity of the fluorescence was calculated using ImageJ software (version 1.53a). Similar results were obtained from three independent experiments. Data are presented as mean ± SD (one-way ANOVA followed by Turkey’s post hoc test). ****, *p* < 0.001 compared with 6-OHDA-only treatment group.

Under pathological conditions, the ROS level is an important parameter of oxidative stress and cell death. Further work measured levels of ROS in 6-OHDA-treated SH-SY5Y cells. As presented in Figure 2, 6-OHDA (100 µM) significantly induced the production of ROS compared with the control treatment, whereas berberine (1, 3, 5, 10 µM) dramatically reduced the accumulation of ROS in a dose-dependent manner. As analyzed using Image J program, berberine doses (1, 3, 5, 10 µM) significantly reduced ROS production by 75.9%, 71.3%, 43.6%, and 34.6%, respectively, compared with cells treated with 6-OHDA only.

These results suggest that berberine could correct the mitochondrial dysfunction induced by 6-OHDA.

### 2.3. Berberine Regulates Mitochondrial Protein Markers and Autophagic Flux Balance Protein Markers in 6-OHDA-Induced SH-SY5Y Cells

**Figure 3:**
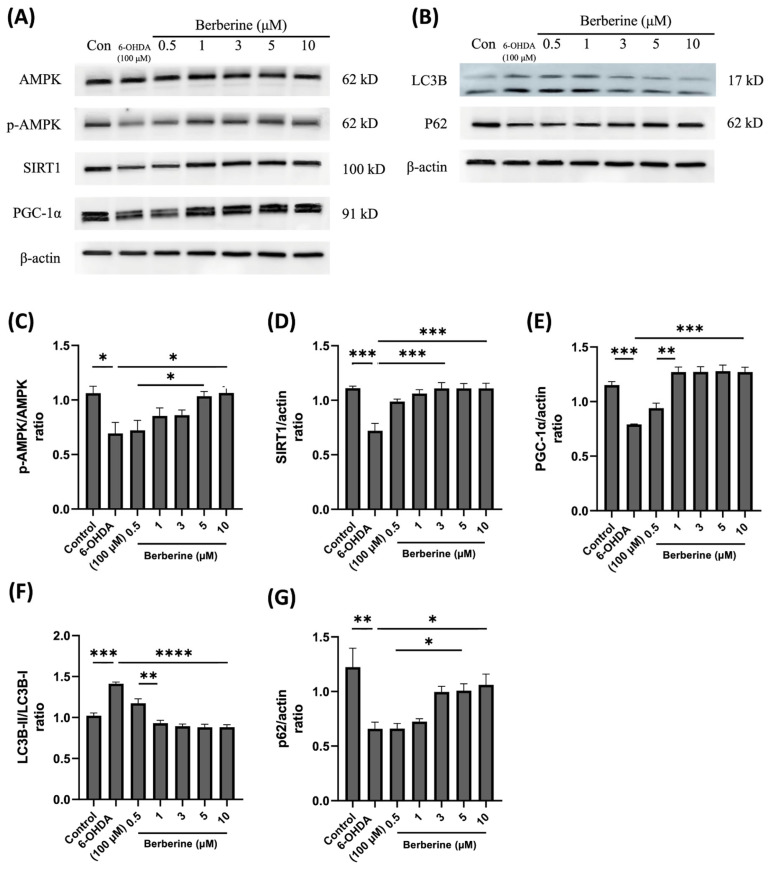
Effects of berberine on AMPK-mediated mitochondrial health and the autophagic flux balance signaling pathway inhibited by 6-OHDA in SH-SY5Y cells. (**A**) Levels of AMPK phosphorylation, and the expression of SIRT1 and PGC-1α proteins. (**B**) The expression of LC3B and p62 proteins. (**C**) p-AMPK/AMPK quantitative graph. (**D**) SIRT1/actin quantitative graph. (**E**) PGC-1α/actin quantitative graph. (**F**) LC3B-II/LC3B-I quantitative graph. (**G**) p62/actin quantitative graph. SH-SY5Y cells were treated with berberine (0.5, 1, 3, 5, 10 µM) for 24 h and then incubated with 100 µM 6-OHDA for a further 6 h. The intensity of the band was calculated using Image J software. β-actin acted as an internal control. Similar results were obtained from three independent experiments. All results were expressed as mean ± SD (one-way ANOVA followed by Turkey’s post hoc test) * *p* < 0.05, ** *p* < 0.01, *** *p* < 0.005, **** *p* < 0.001 6-OHDA-only treatment group compared with control group and Berberine 10 µM group).

To investigate the impact of berberine on the expression of mitochondrial protein markers and autophagic flux balance protein markers, SH-SY5Y cells were subjected to pre-treatment with berberine at various concentrations (1, 3, 5, 10 µM) for a duration of 24 h. Subsequently, cells were exposed to 100 µM 6-OHDA for an additional 6 h, following which expression levels of the relevant proteins were evaluated. As depicted in Figure 3, the administration of berberine effectively counteracted fluctuations in the expression of the p-AMPK, SIRT1, PGC-1α, LC3B, and p62 proteins induced by 6-OHDA. These findings suggest that berberine has protective effects against oxidative stress caused by 6-OHDA. This might be primarily stemming from activation of the AMPK-mediated signaling pathway, which is closely linked to the activation or inhibition of SIRT1 and PGC-1α. This result might also be closely associated with the activation or inhibition of LC3B and p62. In conclusion, our study demonstrates that berberine exhibits a significant ability to modulate the expression of mitochondrial protein markers and autophagic flux balance protein markers in SH-SY5Y cells. This modulation is particularly evident in the context of oxidative stress induced by 6-OHDA. The activation of the AMPK-mediated signaling pathway, along with its association with SIRT1 and PGC-1α, appears to play a crucial role in mediating protective effects of berberine. These findings can enhance our understanding of potential therapeutic applications of berberine in alleviating oxidative stress-related neuronal damage.

### 2.4. Berberine Ameliorates 6-OHDA-Induced Motor Impairments

**Figure 4:**
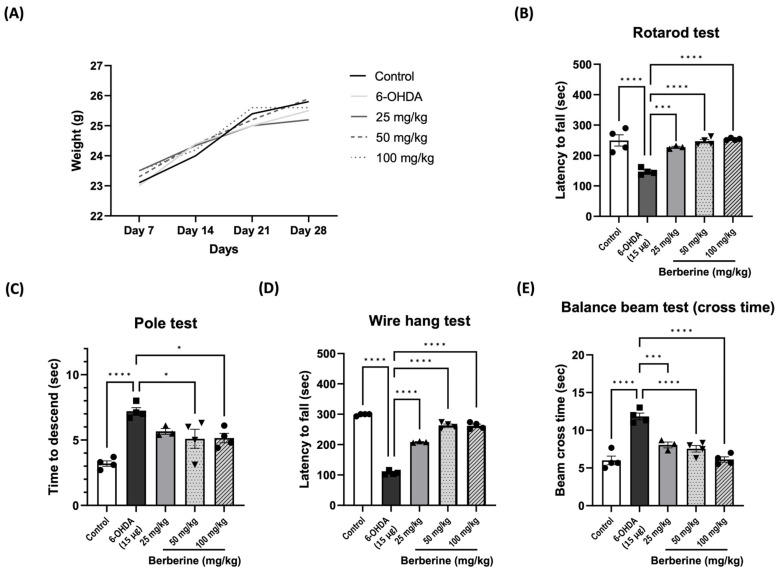
Berberine ameliorated 6-OHDA-induced motor impairments. (**A**) The effects of berberine on mice body weight from day 7 to day 28 of age. (**B**) Latency (sec) to fall in the rotarod test. (**C**) Time (sec) to descend in the pole test. (**D**) Latency (sec) to fall in the wire hang test. (**E**) Beam cross time (sec) in the balance beam test; the data are presented as the mean ± SD (one-way ANOVA followed by Turkey’s post hoc test). * *p* < 0.05, *** *p* < 0.005, **** *p* < 0.001. 6-OHDA-only treatment group compared with control group and berberine treatment group. The circles, squares, and triangles in each graph represent individual values for that group.

To assess the potential of berberine in ameliorating PD-like symptoms, we established 6-OHDA-induced PD models. Behavioral tests were conducted on the 7th and 14th days after the last injection of 6-OHDA. Movement disorders, motor coordination, and spontaneous locomotor activity were evaluated using an accelerating rotarod, pole test, wire hang test, and balance beam test, respectively. The results showed that oral administration of berberine did not have an impact on mouse body weight (Figure 4A). However, berberine effectively prevented and improved motor impairments. Compared to the control group (PBS only) and 6-OHDA-only groups, mice pre-treated with berberine exhibited a reduced time for climbing down the pole and longer latencies in falling from the accelerating rod. Additionally, they spent more time on the wire but less time crossing the beam (Figure 4B–E). All comparisons showed statistical significance.

### 2.5. Modulation of Mitochondrial Protein Markers and Autophagic Flux Balance Protein Markers by Berberine in 6-OHDA-Induced Mouse Model

**Figure 5:**
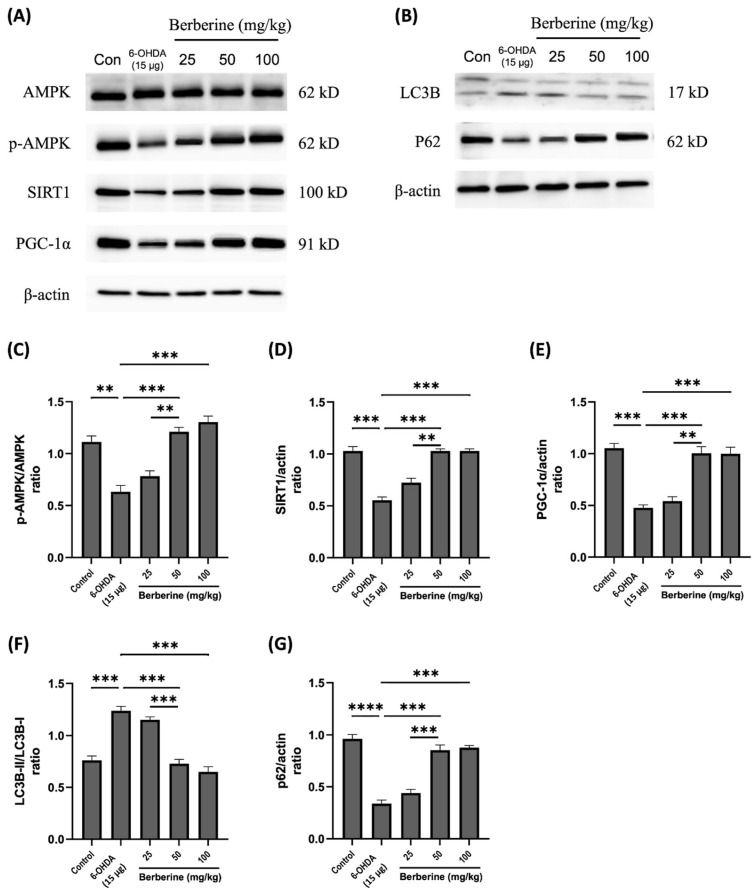
Effects of berberine on 6-OHDA inhibited AMPK-mediated mitochondrial dynamics and autophagic flux balance in animal model. (**A**) Levels of AMPK phosphorylation and expression of SIRT1 and PGC-1α proteins. (**B**) Expression levels of LC3B and p62 proteins. (**C**) p-AMPK/AMPK quantitative graph. (**D**) SIRT1/actin quantitative graph. (**E**) PGC-1α/actin quantitative graph. (**F**) LC3B-II/LC3B-I quantitative graph. (**G**) p62/actin quantitative graph. C57BL/6 mice were administered with berberine orally at different doses (25, 50, 100 mg/kg) for four weeks. Subsequently, mice were injected with 6-OHDA at a dose of 15 μg, and berberine was administered orally. Proteins were isolated from the right striatum tissue, which was the site of 6-OHDA injection. The intensity of the band was calculated using ImageJ software (version 1.53a). β-actin was used as an internal control. Similar results were obtained from three independent experiments. All results are expressed as mean ± SD (one-way ANOVA followed by Turkey’s post hoc test). ** *p* < 0.01, *** *p* < 0.005, **** *p* < 0.001. Berberine treatment group was compared with the control group and the 6-OHDA-only treatment group.

To investigate the influence of berberine on the expression of mitochondrial protein markers and autophagic flux balance makers, a 6-OHDA-induced mouse model was used. Mice were pre-treated with berberine at different concentrations (25, 50, 100 mg/kg) for 6 weeks, followed by injection of 15 μg 6-OHDA to the right striatum for an additional one week. Expression levels of the relevant proteins were then evaluated. As shown in Figure 5, berberine administration effectively mitigated expression levels of p-AMPK, SIRT1.

PGC-1α, LC3B, and p62 proteins that were disrupted by 6-OHDA. These results suggest that berberine’s protective effects against oxidative stress caused by 6-OHDA are primarily mediated through activation of the AMPK-mediated signaling pathway, which is closely linked to the regulation of SIRT1, PGC-1α, LC3B, and p62. In conclusion, our study demonstrates that berberine has a significant ability to modulate the expression of mitochondrial protein markers and autophagic flux balance markers in a 6-OHDA-induced mouse model. This modulation is particularly evident in the context of oxidative stress induced by 6-OHDA. Activation of the AMPK-mediated signaling pathway, along with its associations with SIRT1 and PGC-1α and interactions with LC3B and p62, plays a crucial role in mediating protective effects of berberine. These findings contribute to our understanding of potential therapeutic applications of berberine in mitigating oxidative stress-related neuronal damage.

### 2.6. Using Mouse Serum for In Vivo Toxicity Evaluation of Berberine: Measuring AST and ALT Levels

**Figure 6:**
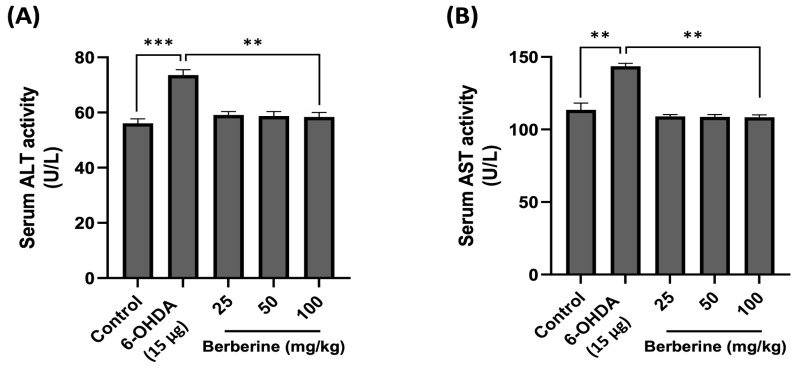
Effects of berberine on hepatotoxicity. (**A**) Serum ALT activity level. (**B**) Serum AST activity level. Liver toxicity was evaluated based on serum ALT/AST activity levels. Blood samples were obtained via orbital blood collection prior to sacrifice, followed by serum separation through centrifugation. ALT and AST levels were quantitatively assessed using an ELISA kit. Optical density (O.D) values were then measured at 450 nm. Data are presented as mean ± SD (one-way ANOVA followed by Turkey’s post hoc test). ** *p* < 0.01, *** *p* < 0.005 compared to the 6-OHDA-only treatment group.

The toxicity and potential impact of berberine on physiological parameters were then assessed in vivo. AST and ALT levels in mouse serum were measured using ELISA kits to determine hepatotoxicity after berberine administration. Mouse serum samples were obtained by centrifuging blood collected through orbital blood collection after completing all behavioral experiments. As shown in Figure 6, AST and ALT levels in the experimental group treated with 6-OHDA only were increased compared to those in the control group. However, the group with berberine intake showed AST and ALT levels similar to those in the control group. These results suggest that not only does berberine not have hepatotoxicity, but it also contributes to detoxification.

## 3. Discussion

PD is a neurodegenerative disorder that is often associated with severe oxidative stress, mitochondrial dysfunction, and changes in protein expression [42]. Recent evidence has suggested that the normal constant process of autophagy is essential for neuronal survival and that its disruption can lead to neurodegeneration. Autophagy impairment has been implicated in the development of PD [43,44]. Natural compounds such as antioxidants, anti-inflammatory agents, and anti-apoptotic agents with neuroprotective properties have been widely used due to their presence in readily available raw materials with minimal toxicity [45,46]. Berberine is an isoquinoline alkaloid found in various edible sources. It has been shown to be able to counteract oxidative stress, inflammation, and neuronal disease [47,48]. In this study, we investigated the neuroprotective effects of berberine on 6-OHDA-induced neurotoxicity in both SH-SY5Y cells and an animal model. We also determined the mechanism of action underlying berberine’s neuroprotective effects.

Previous reports have revealed that 6-OHDA is a selective catecholaminergic neurotoxin commonly used in experimental cell and animal models of PD. 6-OHDA can be absorbed by dopamine transporters, leading to the production of excessive ROS by inhibiting mitochondrial electron transport chain complexes I and IV [49]. In long-lived non-mitotic cells such as neurons, oxidative stress is caused by the excessive production of mitochondrial ROS or damage to the antioxidant defense system, leading to mitochondrial dysfunction and cell death cascade initiation. Excessive ROS and redox homeostasis changes in mitochondria have been linked to PD [50]. In this experiment, we found that harmful effects of 6-OHDA could be effectively relieved by berberine pretreatment. Based on previous experiments [51], we conducted experiments with a wider range of berberine concentrations (0.5–30 µM) and found that orally administered berberine can cross the BBB and reach the brain [52,53]. We also checked the literature and conducted experiments with berberine at oral doses of 25, 50, and 100 mg/kg [54]. Numerous studies have demonstrated the efficacy of berberine in activating AMPK. When AMPK is activated, downstream proteins such as SIRT1 and PGC-1α known to play a crucial role in maintaining mitochondrial health and autophagic flux balance can regulate the corresponding signaling pathway [40,55,56]. SIRT1 and PGC-1α are essential regulators of the mitochondrial signal transduction pathway, while LC3B and p62 are key regulators of the autophagic signal transduction pathway associated with AMPK [57,58]. The current results showed that berberine reversed effects of 6-OHDA by activating AMPK expression and upregulating SIRT1 and PGC-1α proteins. Considering that the accumulation of ROS could lead to mitochondrial dysfunction, autophagy impairment, and cell death, we further examined effects of berberine on ROS production, mitochondrial dysfunction, and autophagy impairment in 6-OHDA-treated SH-SY5Y cells and a 6-ODHA-induced animal model. Our data revealed that 6-OHDA obviously promoted ROS generation, whereas these effects were efficiently blocked by pretreatment with berberine. These results indicate that berberine can improve 6-OHDA-induced oxidative stress, which otherwise would result in neurotoxicity, mitochondrial dysfunction, and autophagy impairment-related protein upregulation.

Animal experiments were conducted based on the results of the cell experiments. Following a 4-week oral administration of berberine, its effectiveness was assessed through behavioral experiments conducted with a mouse model induced with 6-OHDA. The rotarod test, pole test, wire hang test, and balance beam test were utilized to evaluate motor skills associated with endurance, balance, and hand grip strength. The findings indicated an enhancement in exercise capacity across all tests, implying that berberine has the potential to alleviate pathological symptoms associated with PD. Consistent with our in vitro findings, in vivo results confirmed that berberine exerted its effects by activating AMPK and upregulating key downstream targets, including SIRT1, PGC-1α, LC3B, and P62. These results indicate that berberine can modulate similar molecular pathways in both cellular and animal models. Furthermore, AST and ALT analyses revealed no significant hepatotoxicity of berberine in mice. Favorable effects observed for body weight further support the in vivo safety profile of berberine.

Taken together, these findings suggest that berberine not only can alleviate pathological features of PD but also can exert disease-modifying effects. Given its efficacy and low toxicity, berberine represents a promising therapeutic candidate for treating PD and potentially other neurodegenerative disorders.

## 4. Materials and Methods

### 4.1. Chemicals and Antibodies

Berberine (purity > 98%), 6-hydroxydopamine hydrochloride, thiazolyl blue tetrazolium bromide, and dimethyl sulfoxide (DMSO) were purchased from Sigma–Aldrich (St. Louis, MO, USA). Fetal bovine albumin and Dulbecco’s modified Eagle’s medium (DMEM) were purchased from Hyclone (Logan, UT, USA). Penicillin–streptomycin (10,000 U/mL) was purchased from Gibco (Maryland, MD, USA). RIPA buffer was purchased from iNtRON Biotechnology (Seoul, Republic of Korea). Protein assay kit I was purchased from Bio-Rad Laboratories (Hayward, CA, USA). Anti-AMPK, anti-phospho AMPK, anti-LC3B, anti-rabbit IgG-horseradish peroxidase (HRP) conjugate, and anti-mouse HRP antibodies were purchased from Cell Signalling Technology (Danvers, MA, USA). Anti-PGC-1α, anti-SIRT1, anti-SQSTM1/p62 antibodies, and mouse ALT/AST ELISA kit were purchased from Abcam PLC (Cambridge, UK). Beta-actin was purchased Santa Cruz Biotechnology (Santa Cruz, CA, USA). Polyvinylidene fluoride (PVDF) was purchased from Merck Millipore (Billerica, MA, USA). Enhanced chemiluminescence (ECL) solution was purchased from Amersham Pharmasha Biotech (Buckingham, UK).

### 4.2. Cell Culture

SH-SY5Y, a human neuroblastoma cell line, was purchased from ATCC and maintained in Dulbecco’s modified Eagle’s medium (DMEM) (Sigma-Aldrich, St. Louis, MO, USA) supplemented with 10% (*v*/*v*) fetal bovine serum (FBS) and 5 mg/mL penicillin/streptomycin in an atmosphere with 5% CO_2_.

### 4.3. Cell Viability Test

The cytotoxicity of berberine to SH-SY5Y cells was evaluated by MTT (3-(4,5-dimethylthiazol-2-yl)-2,5-diphenyl-tetrazolium bromide) assay. SH-SY5Y cells were plated into 96-well plates at 7 × 10^3^ cells per well in 100 µL of media, pretreated with berberine (0.5, 1, 3, 5, 10, 20, 30 µM) [51] for 24 h, and subsequently exposed to 6-OHDA (100 µM) for 6 h. After 10 µL of the MTT labeling reagent (5 mg/mL) was added to each well, cells were incubated at 37 °C for the next 4 h, and then the medium was removed. Blue crystals, which were the metabolized product of MTT, were extracted by DMSO. The absorbance of each sample was measured with a VERSA max microplate reader (Molecular Devices, LLC, San Jose, CA, USA) at a wavelength of 570 nm to estimate the proportion of surviving cells.

### 4.4. Intracellular ROS Detection

SH-SY5Y cells were seeded into 24-well plates at 5 × 10^4^ cells per well and incubated for 24 h. Cells were then subjected to treatment with berberine at different concentrations (1, 3, 5, 10 µM) for 24 h and 6-OHDA (100 µM) for 6 h. Cells were then incubated with 50 mM DCFH-DA for 30 min. DCFH-DA fluorescence was imaged using a Leica DM4000 M fluorescence microscope (Leica Microsystems, Wetzlar, Germany), and fluorescence intensities were quantified using Image J software (version 1.53a; National Institutes of Health, Bethesda, MD, USA).

### 4.5. Western Blotting

SH-SY5Y cells were seeded in 12-well plates at 5 × 10^4^ cells/well. After seeding, they were pretreated with berberine at different concentrations (0.5, 1, 3, 5, 10 µM) for 24 h and subsequently exposed to 6-OHDA (100 µM) for 6 h. Cells were collected, and RIPA lysis buffer was added. The mixture was then centrifuged at 22,132× *g* at 4 °C for 15 min (Centrifuge 5910 Ri, Eppendorf, Germany). The concentration of protein was then determined using a BCA protein assay kit (Bio-Rad Laboratories, Hercules, CA, USA). Proteins (20 µg) were then separated by 10% SDS-polyacrylamide gel electrophoresis. Proteins were transferred onto PVDF membranes. After blocking with 5% skimmed milk at room temperature for one hour, membranes were incubated with primary antibodies at 4 °C overnight followed by incubation with secondary antibodies at room temperature for one hour. Protein bands were captured with an ECL detection system (Amersham Pharmacia Biotech, Buckinghamshire, UK). Results were analyzed using ImageJ software (version 1.53a; National Institutes of Health, Bethesda, MD, USA).

### 4.6. Animals

**Figure 7:**
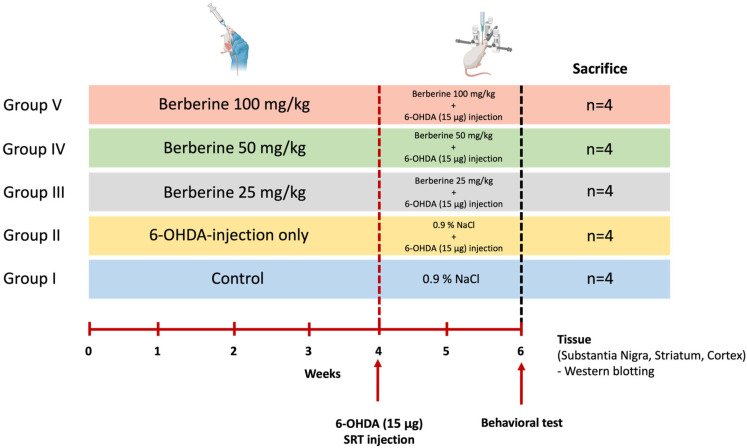
Schematic representation of the study design for the animal model experiment. Age- and gender-matched C57BL/6 mice were divided into five groups. Control group [Group I] and group II mice (*n* = 4 each) received saline (0.9% NaCl) daily by oral gavage for 6 weeks. Groups III, IV, and V (*n* = 4 each) received 25, 50, and 100 mg/kg body weight berberine daily by oral gavage for 6 weeks. Groups II, III, IV, and V received 6-OHDA (15 μg) injection into the right striatum at week 4. After each treatment, behavioral experiments were conducted. After completing the experiments, the mice were sacrificed, and brain tissues were collected to perform various analyses.

Twenty 7-week-old male C57BL/6 mice raised in a sterile facility were purchased from KOATECH (Pyeongtaek, Gyeonggi-do, Republic of Korea) and were allowed to rest for one week before conducting the experiment. We used only the minimum number of animals we thought would yield statistics. These mice showed normal phenotypes and behavior. Mice had an average weight of 30 g at the day of surgery. Animals were randomly divided into five groups of four each without any specific criteria (Control, 6-OHDA-only, Berberine 25, 50, 100 mg/kg) (*n* = 4 per group, total *n* = 20). During the whole experiment, mice were housed under a 12 h/12 h light/dark cycle. They had free access to food and water. All animal experiments were approved by the Institutional Animal Care and Use Committee (IACUC) of BIORCHESTRA (Approval code: BOIACUC-20230404-0002, Approval date: 4 April 2023) and conducted in accordance with the relevant guidelines and regulations. Animal experiments were conducted by administering berberine (25, 50, and 100 mg/kg) [52,53,54] daily for 6 weeks, and 6-OHDA was injected at week 4. Experiments were then conducted two weeks later (Figure 7). Data from all experimental animals were included without any specific criteria.

### 4.7. 6-OHDA Injection

Sixteen mice were anaesthetized with 4% isofluorane (ISOTROY, Troikaa, Pharmaceuticals Ltd., Gujarat, India) in a 2:1 oxygen/nitrous oxide mixture. They were placed in a stereotaxic mouse frame and exposed to 1–2% isofluorane. After dissolving 6-OHDA (Sigma-Aldrich, Stockholm, Sweden) in 0.02% ice-cold ascorbate/saline solution to have a concentration of 5 mg/mL, the solution was used within 3 h [59]. Desipramine (2.5 mg/mL) was injected intraperitoneally (I.P.) at 30 min before surgery. Toxin was injected as a single bolus of 1 µL into the STR (striatum) at the following coordinates (relative to bregma): anterior–posterior (A/P) = + 0.9, medio-lateral (M/L) = − 2.2, and dorso-ventral (D/V) = − 2.5 (from the dura) with a flat skull position [60]. Injections were made using a glass capillary with an outer tip diameter of 50 µM attached to a 10 µL Hamilton syringe at a rate of 0.5 µL over 1 min for 6 min (total of 15 µg/mouse) and then slowly removing the capillary.

### 4.8. Behavioral Test

Behavioral assessments were performed at seven and fourteen days after the surgery.

### 4.9. Rotarod Test

Mice were trained on the rotarod apparatus (3 cm rod diameter, Ugo Basile S.R.L., Gemonio, Italy) at a fixed speed of 10 rpm for 600 s once daily for three consecutive days. Six weeks after berberine injection, performance on the rod was evaluated at a constant acceleration rate of 4–40 rpm in 300 s. Two consecutive trials were performed at 60 min intervals [61].

### 4.10. Pole Test

The pole test is a simple behavior test used to assess motor dysfunction after stroke [62]. Mice were placed in the top of a 60 cm vertical pole with a diameter of 1 cm. The pole was placed in the home cage so that mice could descend to the floor of cage. Recording was started when the animal began the turning movement. The time to turn completely downward and total time to descend to the floor were recorded. When the animal paused while descending, the trial was repeated.

### 4.11. Wire Hang Test

For the wire hang test of motor coordination, mice were tested on 2 mm thick and 55 cm long taut metal wires [63]. The custom-built wire hang apparatus consisted of a black polystyrene box that was 60 cm long into which mice could fall. The latency of mouse to fall from the wire after being suspended was recorded, and the longest suspension time was measured in three trials per mouse.

### 4.12. Balance Beam Test

Mice were tested on a 0.5 cm wide, 1 m long balance beam apparatus. The balance beam consisted of a transparent Plexiglas structure that was 50 cm high with a dark resting box at the end of the runway. Mice were trained on the beam three times in the morning, allowing for a resting inter-trial period of at least 15 min. Mice were left in a dark resting box for at least 10 s before being placed back in their home cages. Mice were then re-tested in the afternoon, at least 2 h after the training session. During test sessions, mice performance was recorded. The test consisted of three trials with a resting inter-trial period of at least 10 min. The number of total paw slips was calculated manually for the last of three tests [64].

### 4.13. Hepatotoxicity Test

Plasma was obtained for enzymatic determination of alanine aminotransferase (ALT) and aspartate aminotransferase (AST) levels using a Mouse ALT/AST ELISA kit (Abcam PLC, Cambridge, UK). The ALT activity was expressed in units per liter, with one unit defined as the amount of enzyme that catalyzed the conversion of 1 µmol of L-alanine into 2-oxoglutarate per min. The AST activity was expressed in units per liter, with one unit defined as the amount of enzyme that catalyzed the conversion of 1 μmol of L-aspartate into 2-oxoglutarate per min.

### 4.14. Statistical Analysis

All results are expressed as mean ± standard deviation (SD). All data analyses were implemented using one-way analysis of variance (ANOVA) in Prism 9 (IBM). *p*-values of less than 0.05 were regarded as being statistically significant.

## 5. Conclusions

This study demonstrates that berberine effectively attenuates 6-OHDA-induced neurotoxicity by reducing oxidative stress, restoring mitochondrial function, and improving autophagic flux in both cellular and animal models of Parkinson’s disease. These neuroprotective effects are mediated, at least in part, through the activation of the AMPK–SIRT1–PGC-1α signaling axis. Behavioral assessments further confirmed the therapeutic potential of berberine in improving motor deficits, while the absence of significant hepatotoxicity supports its safety in vivo. Taken together, these findings suggest that berberine not only alleviates the pathological symptoms of PD but may also offer disease-modifying benefits. Given its pharmacological efficacy and favorable safety profile, berberine holds promise as a potential therapeutic agent for PD and other neurodegenerative disorders.

## Data Availability

The datasets used and/or analyzed during the current study are available from the corresponding author on reasonable request. Some data may not be available owing to privacy or ethical restrictions.
